# Mob2 Insufficiency Disrupts Neuronal Migration in the Developing Cortex

**DOI:** 10.3389/fncel.2018.00057

**Published:** 2018-03-12

**Authors:** Adam C. O’Neill, Christina Kyrousi, Melanie Einsiedler, Ingo Burtscher, Micha Drukker, David M. Markie, Edwin P. Kirk, Magdalena Götz, Stephen P. Robertson, Silvia Cappello

**Affiliations:** ^1^Department of Women’s and Children’s Health, University of Otago, Dunedin, New Zealand; ^2^Helmholtz Center, Institute of Stem Cell Research, Munich, Germany; ^3^Max Planck Institute of Psychiatry, Munich, Germany; ^4^Helmholtz Center Munich, Institute of Diabetes and Regeneration Research, Garching, Germany; ^5^Helmholtz Center, iPSC Core Facility, Munich, Germany; ^6^Department of Pathology, University of Otago, Dunedin, New Zealand; ^7^Sydney Children’s Hospital, University of New South Wales and New South Wales Health Pathology, Randwick, NSW, Australia; ^8^Physiological Genomics, Biomedical Center, Ludwig-Maximilians-University, Munich, Germany; ^9^Excellence Cluster of Systems Neurology (SYNERGY), Munich, Germany

**Keywords:** Mob2, Hippo pathway, periventricular heterotopia, cortical development, exome sequencing

## Abstract

Disorders of neuronal mispositioning during brain development are phenotypically heterogeneous and their genetic causes remain largely unknown. Here, we report biallelic variants in a Hippo signaling factor—*MOB2*—in a patient with one such disorder, periventricular nodular heterotopia (PH). Genetic and cellular analysis of both variants confirmed them to be loss-of-function with enhanced sensitivity to transcript degradation via nonsense mediated decay (NMD) or increased protein turnover via the proteasome. Knockdown of *Mob2* within the developing mouse cortex demonstrated its role in neuronal positioning. Cilia positioning and number within migrating neurons was also impaired with comparable defects detected following a reduction in levels of an upstream modulator of Mob2 function, Dchs1, a previously identified locus associated with PH. Moreover, reduced Mob2 expression increased phosphorylation of Filamin A, an actin cross-linking protein frequently mutated in cases of this disorder. These results reveal a key role for Mob2 in correct neuronal positioning within the developing cortex and outline a new candidate locus for PH development.

## Introduction

During mammalian brain development, new neurons are produced from germinal niches lining the lateral ventricles of the cerebral cortex. From here, neurons must migrate to the outer cortical plate (CP), where they establish connections, contributing to normal brain function (Götz and Huttner, 2005). Broadly grouped, these crucial processes begin with neurogenesis, followed by neuronal migration, differentiation and spatial organization to establish connectivity (Komuro and Rakic, 1998; Kriegstein and Noctor, 2004). Disruption of these developmental processes can lead to a range of neurodevelopment conditions, the understanding of which can inform on the mechanisms governing normal brain development.

Periventricular nodular heterotopia (PH) is a phenotypically heterogeneous condition of cortical development characterized by a failure of neurons to populate the outer cortex of the brain, resulting in heterotopic positioning along their sites of origin—the margins of the lateral ventricles (Guerrini and Dobyns, 2014). Although variants in eight genes have been shown to cause PH, these only account for ~26% of sporadic cases (Fox et al., 1998; Sheen et al., 2004; Parrini et al., 2006; Cappello et al., 2013; Conti et al., 2013; Broix et al., 2016; Alcantara et al., 2017; Oegema et al., 2017). An important etiological role for genetics is, however, hypothesized in many of the remaining individuals with PH (Mandelstam et al., 2013). Mutations in the gene *FLNA*, encoding the F-actin binding protein FilaminA, represent the main cause for established monogenic forms of PH accounting for almost all familial X-linked presentations (Fox et al., 1998). A rare autosomal recessive form of PH with associated microcephaly has also been described in six individuals (Sheen et al., 2004; Bardón-Cancho et al., 2014). The causative gene associated with this phenotype is *ARFGEF2*, with all mutations to date being loss-of-function. *In vitro* studies have shown that *ARFGEF2*, encoding ADP-ribosylation factor guanine nucleotide exchange factor 2 (or BIG2), regulates vesicle formation from the Golgi and trafficking to the cellular surface. BIG2 is also reported to regulate the transport of FLNA to its appropriate cellular compartment (Shin et al., 2005).

Mutations in the atypical cadherin receptor ligands *FAT4* and *DCHS1* have also been shown to cause PH (Cappello et al., 2013). FAT4 and DCHS1 function upstream of the MST kinase (aka Hippo) of the Hippo signaling pathway to control two main functions—cellular polarity and proliferation; with nuclear YAP promoting cell proliferation. Although much interest in recent years has focused on the function of the MST kinases and their downstream effectors—NDR1/2 and Yap (Hergovich, 2012), the heterotopic neurons produced upon FAT4 and DCHS1 knockdown could be mitigated by the simultaneous knockdown of Yap (Cappello et al., 2013). These results suggested that disrupted proliferation was the dominant mechanism for the altered neuronal distribution. However, a role for NDR1/2, a separate branch of the Hippo signaling pathway, could also be involved, but as yet, this possibility remains unexplored. Most recently, missense mutations leading to substitutions in the HECT domain of the E3 ubiquitin ligase, NEDD4L, were also implicated in the cause of PH with *in vivo* mouse studies highlighting dysregulation of mTOR and AKT signaling pathways (Broix et al., 2016).

In this study, we hypothesized that existing knowledge of the genetic pathways underpinning PH could be exploited to prioritize variants found in the genes of patients with PH by exome sequencing. Although such genes fall short of proof-of-pathogenicity on genetic grounds, the aim of this strategy is to identify candidate genes with experimentally validated deleterious biallelic variants for more in-depth targeted re-sequencing analyses that further test their causative potential of PH.

## Materials and Methods

### Subject Ascertainment and Ethical Approval

All study participants were ascertained by physician-initiated referral and consented to participate under a University of Otago consent protocol. Ethical approval was obtained from the Southern Regional Ethics Committee O03/016 and the New Zealand Multicenter Ethics Committee MEC08/08/094.

### Whole-Exome Sequencing

Whole-exome sequencing was carried out by Otogenetics Corporation (Norcross, GA, USA). Sequencing libraries were prepared from genomic DNA extracted from leukocytes of parents and patients using Wizard^®^ Genomic DNA Purification Kit (Promega, Cat. A1620) following the manufacturer’s instructions. Library DNA was exome enriched using the Agilent SureSelect Human All Exon V4+UTRs capture kit, and sequenced on an Illumina Hiseq2000, Illumina, San Diego, CA, USA using 100 bp paired-end reads. Alignment of the sequenced DNA fragments to the Ensembl Genome Browser human genome assembly (GRCh37) was carried out using the Burrows-Wheeler Aligner (MEM algorithm) v.0.7.12. After alignments were produced for each individual separately, the data was locally realigned around indels followed by base quality score recalibration consistent with Genome Analysis Tool Kit (GATK) Best Practices (version 3.4–46; Broad Institute). Duplicate reads were removed using PICARD (version 1.140; Broad Institute). Individual variant calling was undertaken using the GATK HaplotypeCaller, followed by joint genotyping and variant quality score recalibration to produce a multisample variant call format file (vcf).

VCF gene context annotation was added using SnpEff v.4.1L. Allele frequencies were obtained from 1000 Genomes Project phase 1, NHLBI GO Exome Sequencing Project (ESP) ESP6500 and the Exome Aggregation Consortium (ExAC) via the GATK VariantAnnotator. All alignments with loci bearing putative *de novo* variants were extracted from the multisample VCF using GATK SelectVariants and SnpSift v.4.1L (SnpEff) that met the following criteria: (1) the read depth should be ≥8 within the patient; (2) at least 20% of the reads should carry the alternate allele; (3) <5% of the reads in either parent should carry the alternative allele; (4) at least two alleles must be observed in the proband; (5) the genotype quality (GQ) score for the offspring should be 99; and (6) the normalized, phred-scaled genotype likelihood (PL) scores in both parents for the three possible genotypes 0/0, 0/1 and 1/1, where 0 is the reference allele and 1 is the alternative allele, should be >0, >20 and >20, respectively. Candidate *de novo* variants were also absent from population controls, including a set of 107 internally sequenced controls and the individuals whose single nucleotide variant data are reported in the Genome Aggregation Database (gnomAD; Lek et al., 2016). All candidate *de novo* variants were Sanger sequenced using the relevant proband and parents for confirmation.

Putative recessive variants were extracted from the VCF that met the following criteria: (1) the read depth should be ≥8 or 20 for compound heterozygous or homozygous alternate genotype calls in the patient, respectively; (2) at least 20% and 90% of the reads in the patient should carry the alternate allele for candidate compound heterozygous and homozygous genotypes, respectively; (3) in the parents, at least one individual requires a read depth ≥30; and (4) candidate recessive variants should not be present in population controls, including a set of 107 internally sequenced controls and the individuals whose single nucleotide variant data are reported in the Genome Aggregation Database (gnomAD; Lek et al., 2016). All candidate recessive variants were Sanger sequenced using the relevant proband and parents for confirmation.

### Framework for Identifying a Recessive Candidate for Functional Follow Up Studies

To prioritize validated recessive variants for functional investigations that assess the effect of the various changes we used the following criteria: (1) remove any candidate in an individual already reported to have a *de novo* event; (2) at least one of the recessive variants is required to be a stop gain/loss, canonical splice site, or a small out of frame insertion/deletion; and (3) using each of the remaining candidate gene names as key word, functional studies were mined from the literature described in Pubmed to determine if interactions with pre-established PH loci and their cognate proteins was described. Although not suggesting any given candidate satisfying this criterion will be confirmed as a contributor to PH etiology, in this study one gene—*MOB2*—was identified as having properties warranting further analysis. Once identified, the minor allele frequency cut off was relaxed to 2% in the 65 large cohort to survey for additional variants within this gene. This, however, did not identify any further variants of significant excess to that expected from control estimates within the ExAC and gnomAD datasets (Lek et al., 2016).

### Cell Culture

C2C12, HEK293, patient and control fibroblasts were cultured in DMEM Dulbecco’s Modified Eagles Medium (DMEM; Gibco^®^) supplemented with 10% fetal bovine serum (FBS) and 1% L-glutamine at 37°C in humidified 5% CO_2_.

### Reprogramming of Human Fibroblasts to Induced Pluripotent Stem Cells (iPSCs HMGU1)

Induced pluripotent stem cells (iPSCs) were reprogrammed from human newborn foreskin fibroblasts (CRL-2522, ATCC). 2.5 × 10^5^ NuFF3-RQ IRR human newborn foreskin feeder fibroblasts (GSC-3404, GlobalStem) were seeded per well of a 6-well tissue culture dish with advanced MEM (12491015, Thermo Fisher Scientific) supplemented with 5% HyClone FBS (SV30160.03HI, GE Healthcare), 1% MEM NEAA and GlutaMAX (11140050; 35050061 Thermo Fisher Scientific). On day 1, 70%–80% confluent CRL-2522 fibroblasts were dissociated using 0.25% Trypsin-EDTA (25200056, Life Technologies), counted and seeded on the NuFF3-RQ cells at two different densities: 2 × 10^4^ cells/well and 4 × 10^4^ cells/well. On day 2, the medium was changed to Pluriton Reprogramming Medium (00–0070, Stemgent) supplemented with 500 ng/mL carrier-free B18R Recombinant Protein (03–0017, Stemgent). On days 3–18, a cocktail of modified mRNAs (mmRNAs) containing *OCT4, SOX2, LIN28, C-MYC*, and *KLF* mmRNAs at a 3:1:1:1:1 stoichiometric ratio was transfected daily. For that purpose, the mmRNAs were mixed in a total volume of 105 μL and were combined with a mix of 92 μL Opti-MEM I Reduced Serum Medium and 13 μL Lipofectamine RNAiMAX Transfection Reagent (31985062, Thermo Fisher Scientific) after separate incubation at RT for 15 min. Cells were transfected for 4 h, washed and fresh reprogramming medium supplemented with B18R was added to the cultures. The mmRNAs with the following modifications: 5-Methyl CTP, a 150 nt poly-A tail, ARCA cap and Pseudo-UTP were obtained from the RNA CORE unit of the Houston Methodist Hospital. Five days after the first transfection, the first morphological changes were noticed, while the first iPSC colonies appeared by day 12–15. On day 16, the medium was changed to STEMPRO hESC SFM (A1000701, Thermo Fisher Scientific) for 5 days. Harvesting of the iPSC colonies was performed after 40 min incubation at 37°C with 2 mg/ml Collagenase Type IV (17104019, Thermo Fisher Scientific) solution in DMEM/F12 (31331093, Thermo Fisher Scientific). The iPSCs were plated on γ-irradiated mouse embryonic fibroblasts (MEFs) and grown in STEMPRO hESC SFM for 10 additional passages. After that the iPSCs were further cultured in a feeder-free culture system, using mTeSR1 (05850, StemCell Technologies) on plates coated with LDEV-Free Geltrex (A1413302, Thermo Fisher Scientific). The control iPSC line HMGU1 was generated in the iPSC Core Facility, Helmholtz Center Munich.

### iPSC Culture

iPSCs were cultured at 37°C, 5% CO_2_ and ambient oxygen level on Geltrex coated plates in mTeSR1 medium with daily medium change. For passaging, iPSC colonies were incubated with StemPro Accutase Cell Dissociation Reagent (A1110501, Life Technologies) diluted 1:4 in PBS for 4 min. Pieces of colonies were washed off with DMEM/F12, centrifuged for 5 min at 300× *g* and resuspended in mTeSR1 supplemented with 10 μM Rock inhibitor Y-27632 (2HCl; 72304, StemCell Technologies) for the first day.

### Cerebral Organoid Generation

Cerebral organoids were generated starting from 9000 single iPSCs/well in 96 well tissue culture plates as previously described (Lancaster et al., 2013). Upon their formation, organoids were cultured in 10 cm dishes on an orbital shaker at 37°C, 5% CO_2_ and ambient oxygen level with medium changes twice a week.

### Transfection of Human Cells

Expression constructs were transiently transfected into C2C12, HEK293 and fibroblast cells with Lipofectamine™ 2000 (Invirogen, Cat. # 11668-019) according to the manufacturer’s instructions. siRNA constructs were transiently transfected into human fibroblasts with Lipofectamine RNAiMAX (Invitrogen, Cat. # 13778-075) according to the manufacturer’s instructions.

### Protein Extraction

Cells were lysed after 20 h post transfection in 1× phosphate buffered saline (PBS), 1% (v/v) Triton-X100 and Complete Protease Inhibitor (Roche) on ice for 20 min. Cell debris was then pelleted through centrifugation at 13,000 rpm 4°C for 10 min. Twenty microliter of protein lysate was combined with protein loading dye (final concentration: 50 mM Tris pH 6.8, 10% glycerol, 2% SDS, 6% 2-mercaptoethanol and 1% w/v Bromo blue) and denatured at 95°C for 5 min.

### SDS-Page and Western Blot

Protein lysate was separated by polyacrylamide gel electrophoresis using a mini-PROTEAN Tetra Cell system (BioRad Laboratories, Hercules, CA, USA). The samples were run through on a 7%–10% running gel, depending on the size of the proteins being separated, and a 5% stacking gel. Gel electrophoresis was performed for 1–2 h at 200 V in running buffer (25 mM Tris base, 192 mM Glycine, 0.1% (v/v) SDS), with 4 μL Precision Plus Protein Dual Colour Standard (BioRad, Cat. # 161-0734) loaded to determine protein size and monitor electrophoresis speed. Proteins were transferred to a nitrocellulose membrane (0.25 or 0.45 micron, depending on protein size, Pierce) using the Min Trans-Blot Electrophoretic Transfer Cell (BioRad Laboratories, Hercules, CA, USA) at 25 V and 50 V for 1.5 h each in transfer buffer (25 mM Tris, 0.19 M Glycine, 10% (v/v) isopropanol). The membrane was then blocked in 5% non-fat milk powder in PBS-T (PBS, 0.05% (v/v) Tween20) for 30 min with shaking at 30 rpm (Ratek platform shaker). The appropriate primary antibody was then diluted into fresh 5% (w/v) non-fat milk powder in PBS-T and incubated with the membrane overnight at 4°C and 30 rpm. The following day the membrane was washed once in PBS-T before incubation with secondary antibody to horseradish peroxidase diluted into fresh 5% non-fat milk powder in PBS-T for 1 h at 30 rpm. The membrane was then washed five times for 5 min in PBS-T before detecting bands with SuperSignal West Pico Chemiluminscent Substrate (Pierce, Cat. # 34080) on Kodak BioMax XAR Film size 8 (Kodak, Cat. # 165-1454). Total FLNA (mouse, Abcam, Cat. #ab188350, 1:3000) and phospho-Filamin A (Ser2152; rabbit, Cell Signalling Technology, Cat. #4761S, 1:1000) were used to assess phosphorylated levels of FLNA at site serine 2152.

### Nonsense Mediated RNA Decay

Age-matched control and patient fibroblasts were cultured in T-25 flask (Falcon, Cat. # 353108). Once at 80% confluency, minimal growth media was substituted (DMEM, 1% FCS, 1% L-glutamine) and cells were cultured for a further 12 h. After 12 h incubation media was substituted for DMEM, 10% FCS, 1% L-glutamine and either solvent alone (DMSO) or 150 μg/mL cycloheximide (CHX) in DMSO. Cells were then incubated for a further 4 h after which time media was removed and cells washed with PBS, before RNA isolation. *MOB2* expression was then assayed by RT-qPCR using gene specific primers. House-keeping normalized *MOB2* expression in patient fibroblasts was compared to control DMSO treatment using the delta-delta Ct method.

### Proteasome Inhibition

HEK293 cells plated at 80% confluency in 24-well plates (Falcon, Cat. # 353047) were transiently transfected with a bicistronic vector expressing both the green fluorescent protein (GFP) and wild-type (WT) or mutant (Glu227Lys) *MOB2*. Twenty-four hours after transfection media was removed and cells gently washed with PBS. Media containing DMEM, 10% FCS, 1% L-glutamine and either solvent alone (0.01% DMSO) or the proteasome inhibitor, 1 μM MG132 (Sigma-Aldrich, Cat. # C2211), in DMSO for 24 h. After which point total cell lysates were prepared and subjected to Western blot analysis using MOB2 (mouse, LSBio, Cat. #LS-C184588, 1:5000), GFP (mouse, OriGene, Cat. # TZ150041, 1:5000) and GAPDH (rabbit, Sigma-Aldrich, Cat. # G9545, 1:3000).

### Apoptosis Assay

Age-matched control and patient fibroblasts were cultured in T-25 flask (Falcon, Cat. # 353108). Once at 80% confluency, cells were treated with 0.5% trypsin (Sigma-Aldrich, Cat. # 59418C) and reverse transfection protocol was carried out in a 24-well plate with cells re-plated at the appropriate densities. Media contained DMEM, 10% FCS and 1% L-glutamine and 30 nM siRNA scrambled or against *MOB2* (Sigma-Aldrich, Cat. # SIC001-10NMOL and SASI_Hs01_00177399) pre-mixed in Lipofectamine RNAiMAX (Invitrogen, Cat. # 13778-075) according to the manufacturer’s instructions. Samples not treated with siRNA were only provided the Lipofectamine RNAiMAX mixture. Twenty-four hours after transfection media was removed, cells gently washed with PBS and then placed in DMEM, 10% FCS, 1% L-glutamine and 200 μM camptothecin (Merck-Millipore, Cat. # 208925). Total cell lysates were prepared 0, 8, 15 and 24 h post camptothecin treatment (200 μM) and processed for immunoblotting using cleaved-PARP (mouse, BD-Pharmigen, Cat. # 552596, 1:1000) and GAPDH (rabbit, Sigma-Aldrich, Cat. # G9545, 1:3000).

### Mouse Lines

All the animals used in this work were kept in the animal facility of the Helmholtz Center Munich and Max Planck Institute of Psychiatry. All the experimental procedures were performed in accordance with German and European Union guidelines. Animals were maintained on a 12 h light-dark cycle. The day of vaginal plug was considered as embryonic day 0 (E0). In this study the C57Bl/6J mouse line was used.

### Anesthesia

To perform *in utero* operations, mice were anesthetized by subcutaneous injection of a solution containing: Fentanyl (0.05 mg/kg), Midazolam (5 mg/kg) and Medetomidine (0.5 mg/kg). The anesthesia was terminated with a subcutaneous injection of a solution composed of Buprenorphine (0.1 mg/kg), Atipamezol (1.5 mg/kg) and Flumazenil (0.5 mg/kg).

### *In Utero* Electroporation

Surgery was performed on animals in accordance with the guidelines of Government of Upper Bavaria under licence numbers 55.2-1-54-2532-79-11 and 55.2-1-54-2532-79-2016. E13 pregnant dams were anesthetized and operated on as previously described (Saito, 2006). In brief, the shaved abdomen was opened by caesarean section in order to expose the uterine horns. These were kept wet and warm by continuous application of pre-warmed saline. Endotoxin free vectors—diluted to 1.5 μg/μL—were mixed in Fast green (2.5 mg/μL, Sigma). One microliter of mix was injected into the ventricle with the aid of glass capillaries (self-made with a micropipette puller). DNA was electroporated into the lateral ventricle of the telencephalon with four pulses of 38 mV for 100 ms each. At the end of the entire electroporation procedure, the uterine horns were repositioned into the abdominal cavity, which was then filled with pre-warmed saline. The abdominal wall was closed by surgical sutures (Ethicon, Cat. # K832H). Anesthesia is reversed as described above and animals were monitored appropriately. At E16 (3 days post electroporation (3 dpe)) operated animals were sacrificed by cervical dislocation. Embryos were placed in HBSS (Hank’s Balanced Salt Solution—Gibco, Life Technologies) supplemented with 10 mM Hepes (Gibco, Life Technologies). Embryos were dissected and brains fixed. pSuper-GFP (Oligoengine) were used as the control plasmid. *Mob2* targeting miRNA-expressing constructs (miRNA1 (5′-ctatactgcaggttgatgtgg-3′) and miRNA2 (5′-aataggagcaagcagcaggtg-3′)) were cloned into the pSuper-GFP vector (Blockit, Invitrogen) according to the manufacturer’s instructions. Plasmids were subsequently recombined into PCAGGS destination vector (Invitrogen Gateway^®^-adapted expression vector, Cat. # K493600). shRNAs against *Dchs1* and *Yap* have been described previously (Cappello et al., 2013). To visualize the cilia, we electroporated a plasmid encoding *Arl13b* fused with tagRFP (*Arl13b*-tagRFP). Nucleus-cilia coupling was assessed in migrating neurons in the upper bin (CP2).

### Electroporation of Cerebral Organoids

Cerebral organoids were kept in antibiotic-free conditions prior to electroporation. Electroporations were performed in cerebral organoids at the 44 days stage after the initial plating of the cells and fixed 7 dpe. During the electroporation cerebral organoids were placed in an electroporation chamber (Harvard Apparatus, Holliston, MA, USA) under a stereoscope and using a glass microcapillary 1–2 μL of plasmid DNAs at final concentration of 1 μg/μL was injected together with Fast Green (0.1%, Sigma) into different ventricles of the organoids. Cerebral organoids were subsequently electroporated with five pulses applied at 80 V for 50 ms each at intervals of 500 ms using the Electroportator ECM830 (Harvard Apparatus). Following electroporation cerebral organoids were kept for additional 24 h in antibiotic-free media, and then changed into the normal media until fixation. Cerebral organoids were fixed using 4% PFA for 1 h at 4°C, cryopreserved with 30% sucrose and then stored in −20°C. For immunofluorescence, 16 μm cryosections were prepared.

### Immunofluorescence of Mouse Cortical Tissue

Mouse cortical tissues were fixed in 4% paraformaldehyde for 20 min at 4°C followed by washing in PBS three times 10 min. Tissues were allowed to sink in 30% sucrose overnight and then embedded into molds (Polysciences, Cat. # 18646A-1) using Tissue-Tek (Hartenstein, Cat. # TTEK) and frozen on dry ice. Tissue was then stored at −20°C until it was cryosectioned in 20 μm sections with a Cryostat (Leica). Sections were blocked and permeabilized in 0.25% Triton-X100, 4% normal donkey serum in PBS. Sections were then incubated with primary antibodies in 0.1% Triton-X100, 4% normal donkey serum at the following dilutions: GFP (chick, Aves Lab, Cat. # GFP-1020, 1:500). Sections were incubated overnight at 4°C. The next day slides were washed three times in PBS and then treated as per the manufacturer’s instructions with the appropriate secondary fluorophore antibodies.

### Immunofluorescence of Human Cerebral Organoids

Tissues were processed as per mouse cortical tissue. Before sections were blocked they were post-fixed with 4% PFA for 10 mins, permeabilized in 0.3% Triton-X100 and then blocked with 0.1% Tween, 3% BSA and 10% normal goat serum. Sections then incubated with primary antibodies diluted in blocking solution. GFP (chick, Aves Lab, Cat. # GFP-1020, 1:1000) and RFP (rbb, Rockland 1:1000, Cat. # 600-401-379).

### Quantification and Statistical Analysis

All analyses were performed using at least three different animals of each genotype and the total number of cells counted is shown below each treatment bar in each graph of the figure. Data are represented as mean ± SEM. Statistical significance was assessed using Mann-Whitney U test, exact binomial test or one-way ANOVA with Tukey HSD correction as indicated.

## Results

### Identifying Candidate Loci With Biallelic Variants in Patients With PH for Functional Tests

To begin identifying candidate variants in PH, we analyzed the coding region of the genome (the exome) in 65 patients with the condition and their unaffected parents. Although *de novo* variants have a strong association in neurodevelopmental conditions, to simplify interpretation in this study we restricted our analysis to rare biallelic variants whereby at least one of the altered alleles was required to be a stop gain/loss or a small out of frame insertion/deletion predictive of loss-of-function (materials and methods). Of the 17 rare biallelic genotypes identified in the 65 patients, only five satisfied these criteria (Supplementary Table S1). Functional studies mined from the literature database Pubmed, with each of the five candidate gene names as key word, identified no evidence for any locus that indicated a described functional interaction with pre-established PH loci and their cognate proteins. There was, however, functional evidence for one locus modulating immediate components of the Hippo signaling pathway, through which the protein products of the known PH genes *FAT4* and *DCHS1* signals (Cappello et al., 2013)—*MOB2*.

The MOB family of proteins are both evolutionary conserved and well-defined regulators of the Hippo signaling pathway (Hergovich, 2012). Specifically, the sole identified interacting partners of MOB2 are the kinases NDR1/2 of the Hippo signaling pathway (Hergovich et al., 2005; Kohler et al., 2010; Hergovich, 2012). As per the Online Mendelian Inheritance in Man database (OMIM), variants in the *MOB* family of genes have not been associated with any disease to date.

### Biallelic Variants Reduce *MOB2* Transcript and Protein Levels *in Vitro*

**Figure 1:**
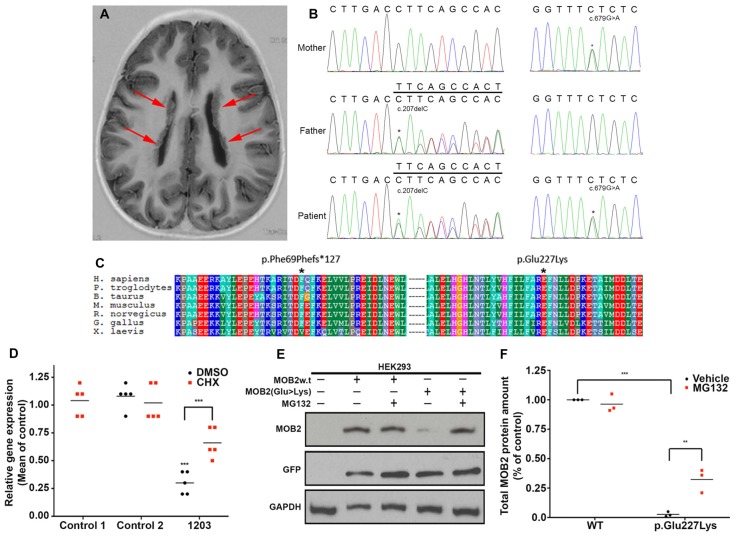
Biallelic variants in *MOB2* affect mRNA and protein stability *in vitro*. **(A)** Brain MRI scan of patient 1203, showing the presence of bilateral periventricular nodular heterotopia (PH; red arrows). **(B)** Sequence traces illustrating the *MOB2* sequence variants c.679G > A, present in mother and patient, and c.207delC, present in father and patient. **(C)** Multiple sequence alignment (ClustalX) of the human MOB2 protein against various species. Columns are colored by conservation and property. ^*^Denotes the positions of the mutated residues—Phe69 and Glu227—substituted by each of the alleles discovered in individual 1203. **(D)** Mean expression of *MOB2* in age-matched controls and individual 1203 (*n* = 5) fibroblasts when treated with vehicle/DMSO (black circles) and cycloheximide (CHX; red squares), as determined by real-time PCR. Expression was normalized against *GAPDH* and *DIMT1* housekeeping genes in the same sample using the relative standard curve method. Values were then subsequently normalized to control 1 DMSO treatment using the delta-delta Ct method. **(E)** HEK293 cells were transiently transfected with a bi-cistronic vector expressing both the green fluorescent protein (GFP) and wild-type (w.t) or mutant (Glu227Lys) *MOB2*. At 24 h after transfection, cells were treated with DMSO, or the proteasome inhibitor, MG132, for 24 h, after which total cell lysates were prepared and subjected to western blot analysis using anti-MOB2, anti-GFP and anti-GAPDH as a loading control. **(F)** Quantifications representing three biological replicates of **(E)** summarizing total MOB2 protein amount with or without MG132 treatment. Lysosomal degradation pathways may account for the remaining degraded MOB2. ^**^P < 0.01; ^***^P < 0.001, Mann-Whitney U test.

Patient 1203, a female, identified here with biallelic variants in *MOB2* was born to healthy non-consanguineous parents. She first presented with epilepsy and learning difficulties. A brain MRI scan revealed bilateral nodular PH of the lateral ventricles (Figure 1A). Specifically, patient 1203 has two variants in *MOB2*, a frameshift variant leading to the introduction of a stop codon and a missense variant (c.207delC, p.Phe69Phefs^*^127; and c.679G > A, p.Glu227Lys; RefSeq NM_001172223 (GRCh37)), respectively (Figure 1B). Neither variant is present within the Genome Aggregation Database (gnomAD; Lek et al., 2016). Sanger validations of the parental genotypes identified they were in *trans* in the proband with each parent being heterozygous for only one of the two variants (Figure 1B). Both variants show patterns of strong evolutionary conservation (Figure 1C).

Analysis of gene constraint using information from the ExAC database (Lek et al., 2016) showed evidence of intolerance against LoF variants in *MOB2*. The constraint metric pLI for this gene in ExAC is 0.64. While scores greater than 0.9 are regarded as indicating extreme intolerance to LoF, the number of predicted LoF variants in ExAC is quite small (7.3, with a single variant actually observed). It is noteworthy that in the much larger gnomAD database, there are only six LoF alleles in total, two of which are located in the final exon, indicating that the resulting transcripts are unlikely to be targeted for nonsense-mediated decay. Taken together, this information indicates that LoF variants in *MOB2* are likely to have a deleterious effect.

The nonsense variant identified in individual 1203, c.207delC, predicts a premature termination codon within exon two and degradation via post-transcriptional mechanisms involving nonsense mediated decay (NMD; Nagy and Maquat, 1998). To formally determine if NMD is activated in response to the c.207delC allele, quantitative RT-PCR of *MOB2* transcripts was carried out on mRNA extracted from patient 1203 fibroblasts. Compared to *MOB2* expression in two age-matched control fibroblast lines, we identified a significant (*P* < 0.001) four-fold reduction in gene expression (Figure 1D). Treating fibroblasts with CHX, a known NMD suppressor (Carter et al., 1995), induced a significant 2.6-fold increase (*P* < 0.001) in *MOB2* transcript relative to vehicle-treated patient cells (Figure 1D).

To investigate any potential detrimental effects conferred by the missense variant (c.679G > A) identified in patient 1203, total protein lysate of HEK293 cells overexpressing the MOB2 constructs (WT or p.Glu227Lys) linked bicistronically to GFP were analyzed by Western blot (Figure 1E). Analysis of total protein lysate 24-h post transfection identified a significant (*P* < 0.001) 10-fold reduction in mutant MOB2 protein harboring the p.Glu227Lys change, relative to WT control (Figure 1F).

In light of these results, we hypothesized that the missense variant, p.Glu227Lys, destabilizes MOB2 resulting in its degradation. In eukaryotic cells, the ubiquitin-dependent proteasome system is the main pathway responsible for selective intracellular protein degradation in response to mis-folding (Nandi et al., 2006). To determine whether this pathway was activated here, HEK293 cells expressing human *MOB2* variants (WT or p.Glu227Lys) were treated with the proteasome inhibitor MG132. Quantitative analysis 24-h post MG132 treatment identified a significant (*P* < 0.01) increase in mutant MOB2 protein harboring the p.Glu227Lys change, relative to vehicle-only treated cells also overexpressing this form of protein (Figures 1E,F). No significant change in total MOB2 abundance was detected between MG132 and vehicle treated HEK293 cells overexpressing the WT protein (Figures 1E,F). No change in transfection efficiency (reported by untagged-GFP encoded on the same bicistronic vector) was identified between any of the treatment groups (Figure 1E).

Direct assessment of the detrimental effect conferred by the missense variant (p.Glu227Lys) in patient 1203 fibroblasts was precluded given the undetectable levels of endogenous MOB2 in control cells. However, reduced MOB2 levels enhance the susceptibility of cells to apoptosis *in vitro* (Kohler et al., 2010). Camptothecin is a potent inducer of apoptosis operating through the inhibition of DNA topoisomerase (Tazi et al., 1997). Thus, to assess altered levels of susceptibility to apoptosis in patient 1203 fibroblasts, cells were treated for 8, 15 and 24 h with camptothecin, after which total protein lysate was isolated and probed with an antibody against cleaved poly ADP-ribose polymerase (PARP)—a protein cleaved directly by caspase-3 upon apoptosis induction (Supplementary Figure S1). Cleaved-PARP was detected 24 h post camptothecin treatment in age-matched control fibroblasts. In contrast, cleaved-PARP was detected 7 h earlier, at 8 h post camptothecin treatment, in patient 1203 fibroblasts (Supplementary Figure S1). This enhanced susceptibility to apoptosis observed in patient 1203 fibroblasts, relative to controls, was comparable to that seen in age-matched control fibroblasts transfected with siRNA targeting *MOB2* (Supplementary Figure S1).

Taken together, these data suggest that both variants identified in *MOB2* of patient 1203 are LoF with NMD and proteasome-dependent protein degradation acting to reduce MOB2 levels.

### *Mob2* Knockdown *in Vivo* Alters Neuronal Distribution Within the Developing Mouse Cortex and Induces Nucleus-Cilia Uncoupling

**Figure 2:**
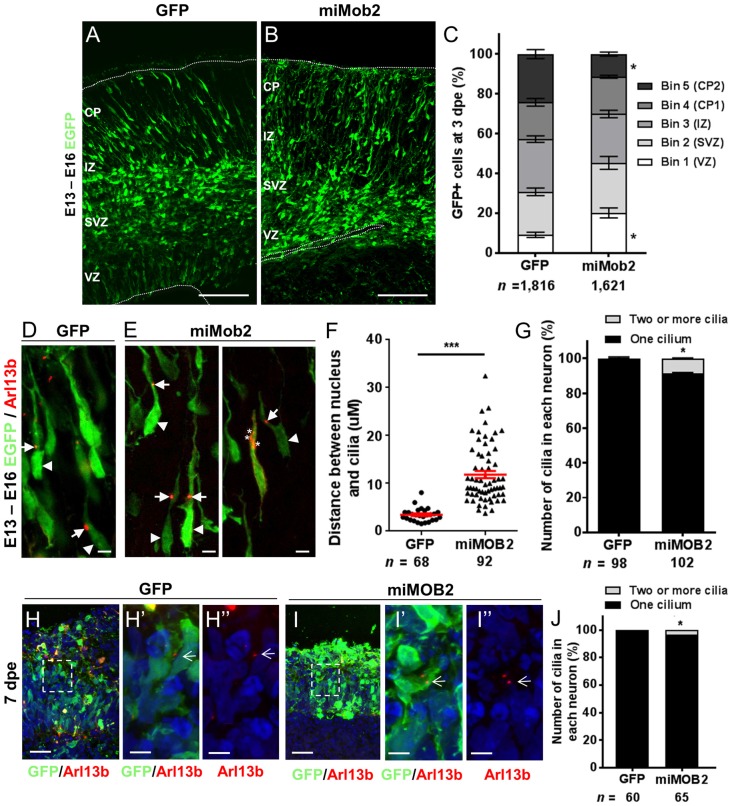
*Mob2* knockdown alters neuronal cell distribution and nucleus-cilia coupling. Coronal micrograph sections of E16 mouse cerebral cortices electroporated at E13 with EGFP/empty vector control **(A)** or miRNAs targeting *Mob2* **(B)**. **(C)** Quantification of the distribution of EGFP-expressing (EGFP+) cells transfected with EGFP/empty vector alone or miRNAs targeting *Mob2* 3 days after electroporation (mean ± SEM). The cortex was subdivided into five equal width bins approximately corresponding to VZ (bin 1), SVZ (bin 2), IZ (bin 3) and CP (bins 4 and 5). CP, cortical plate; IZ, intermediate zone; SVZ, subventricular zone; VZ, ventricular zone; dpe, days post electroporation. At least three embryos were analyzed for each condition. n, total number of GFP+ cells counted per condition. **(D,E)** Representative images of migrating neurons in E16 cortices (3 dpe) electroporated with EGFP/control or miRNA targeting *Mob2* (green) and Arl13b-tagRFP (red) located within bin 5 (CP2). Arrow heads indicate the cell bodies and arrows indicate the cilia labeled by Arl13b-tagRFP. Asterisks show the position of three cilia within a neuron. **(F)** Quantification of the distance between the cilia and nucleus (micrometers) in neurons. Each dot represents a neuron and red lines indicate mean ± SEM. **(G)** Quantification of the number of cilia in each neuron. **(H,I)** Micrographs sections of day 51 human cerebral organoids electroporated at day 44 with EGFP/empty vector control or miRNA targeting *MOB2* (green) and Arl13b-tagRFP (red). White arrows indicate the cilia labeled by Arl13b-tagRFP. **(J)** Quantification of the percentage of cells with more than one cilium of organoids transfected with EGFP/empty vector control (GFP) or miRNAs targeting *Mob2*. Data taken from at least three ventricular structures. **(C,F,G)** Mann-Whitney U test, **(J)** Exact binomial test; ^*^p < 0.05, ^***^p < 0.001. Scale bar represents **(A,B)** 100 μm, **(D,E)** 10 μm, **(H,I)** 25 μm and **(H′,H″,I′,I″)** 5 μm.

Data defining brain structures that express *Mob2* suggest a potential role of this gene in cortical development (Lein et al., 2007; Miller et al., 2014). To test this directly we assessed the effects of knocking *Mob2* down during mouse cortical development. Specifically, we injected a bi-cistronic vector expressing EGFP and validated miRNAs directed against *Mob2* (Supplementary Figure S2) into the ventricular neuroepithelium of embryonic day 13 embryos (E13) using *in utero* electroporation. Three days post electroporation (3 dpe; E16) the electroporated cortices were cross-sectioned into five equally sized bins (approximately corresponding to the different two proliferative zones, intermediate zone (IZ) and upper and lower CP) for analysis. Here, we identified an increased fraction of EGFP-expressing cells in bin 1, corresponding to the ventricular surface (VZ) relative to the vector-only control cortices, with a correspondingly decrease in EGFP-expressing cells in bin 5, corresponding to the outer CP (CP2; Figures 2A–C; *P* < 0.05). No change to the total number of GFP+ cells in either condition was observed. These patterns suggest that reduced *Mob2* levels alter the distribution of neuronal cells within the developing cortex.

The predominant mode of radial neuronal migration within the developing cortex is glial-guided locomotion, regulated by coupled/coordinated movement of the cilia/centrosome and nucleus within the neuron (Marin and Rubenstein, 2003). The kinases NDR1/2 are known modulators of cilia/centrosome dynamics, induced by changes in MOB2 activity *in vitro* (Hergovich et al., 2009). Given this insight and the redistribution patterns observed in cells upon decreased *Mob2* levels *in utero*, we hypothesized that knockdown of *Mob2* could alter cilia/centrosome dynamics. To test this, developing mouse cortices were electroporated with a plasmid encoding the cilia specific protein—Arl13b (Higginbotham et al., 2012)—fused to tagRFP (Arl13b-tagRFP), together with either the plasmid expressing EGFP/control or miRNA targeting *Mob2* bi-cistronically linked to EGFP (Figures 2D–G). In migrating neurons expressing the control vector, the mean distance between the nucleus and cilia (defined by Arl13b-tagRFP) was 3.5 μm, consistent with distances reported in previous studies (Insolera et al., 2014). In migrating neurons expressing hair-pins targeting *Mob2* and co-electroporated with a construct encoding *Arl13b-tagRFP*, however, a significant 3-fold increase in distance between the nucleus and cilia was observed (mean distant 11.84 μm; *P* < 0.001) relative to the control (Figure 2F).

Every migrating neuron contains a primary cilium attached to the centrosome (basal body; Louvi and Grove, 2011). Of the 102 migrating neurons analyzed that express both miRNAs directed against *Mob2* and Arl13b-tagRFP, eight had evidence for the presence of two or more cilia, a significant increase compared to the 98 neurons expressing both the control vector and Arl13b-tagRFP of which two had more than one cilium (Figure 2G; *P* < 0.05). Defects in cilia number were also observed in human cerebral organoid cultures upon *MOB2* knockdown, further suggesting that correct levels of this protein are essential for maintenance of this organelle in human neurons (Figures 2H,H′,H″,I,I′,I″,J). Taken together, these results suggest that knockdown of *Mob2* during E13–E16 of murine brain development, *in utero*, uncouples coordinated movement between the cilium and nucleus and may interfere with the regulation of cilia/centrosome duplication; changes that could ultimately impair effective neuronal migration in a subpopulation of neurons, similarly to what occurs in PH.

### *Dchs1* Knockdown Induces Nucleus-Cilia Uncoupling Within the Developing Mouse Cortex *in Vivo*

**Figure 3:**
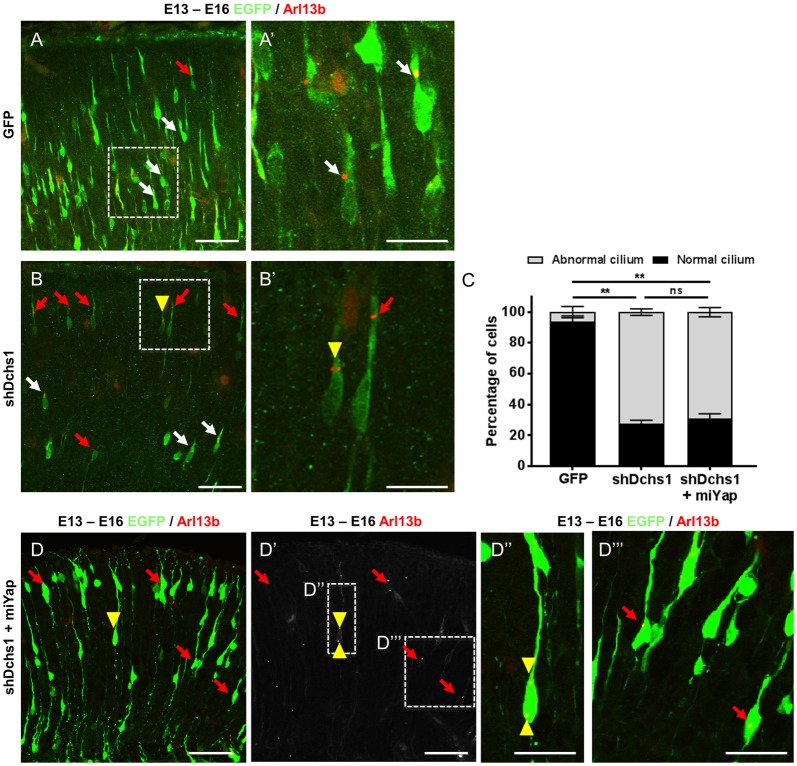
*Dchs1* knockdown in the developing mouse cortex alters nucleus-cilia coupling that cannot be rescued by *Yap* knockdown. **(A,B,D)** Representative images of migrating neurons located within bin 5 (CP2) in E16 cortices (3 dpe) electroporated with EGFP/control, miRNA targeting *Yap* and/or shRNA targeting *Dchs1* (green) and Arl13b-tagRFP (red). White and red arrows indicate migrating neurons with correct or abnormal nucleus-cilia coupling as defined by position, respectively. Yellow arrow head shows migrating neurons with multiple cilia. **(C)** Quantification of the percentage of cells with abnormal nucleus-cilia coupling. Results represented as mean (± SEM). One-way ANOVA with Tukey HSD correction; ^**^p < 0.001; ns, not significant. Scale bar represents **(A,B,D,D′)** 50 μm and **(A′,B′,D″,D‴)** 20 μm.

FAT4 and DCHS1 are directly implicated in the cause of Van Maldergem syndrome, a developmental disorder characterized by PH (Cappello et al., 2013) and function upstream of MOB2 in the Hippo signaling pathway (Hergovich, 2012). Given this, and the altered cellular distribution and nucleus-cilia uncoupling observed upon *Mob2* knockdown in mouse embryonic cortices, we investigated if dysregulation of other genes involved in the regulation of the Hippo pathway and in which mutations were associated with PH also altered these uncoupling events. Indeed, knockdown of *Dchs1* by *in utero* electroporation induced cilia defects in the developing mouse cortex (Figures 3A,A′,B,B′,C). In particular, the position and/or number of cilia in migrating neurons was aberrant upon *Dchs1* downregulation. Strikingly, unlike the altered proliferation phenotype induced by knockdown of this factor (Cappello et al., 2013), this effect could not be remedied by simultaneous reduction of *Yap* expression (Figures 3C,D,D′,D″,D‴). As the Hippo signaling pathway is composed of two arms, the Yap arm and Mob2 arm (Figure 5; Hergovich, 2012), these results suggest that the changes previously observed upon *Dchs1* knockdown *in utero* with respect to proliferation related directly to the regulation of Yap (Cappello et al., 2013). In contrast, defects in nucleus-cilia coupling could be independently determined by interference with Mob2 function in migrating neurons.

**Figure 4:**
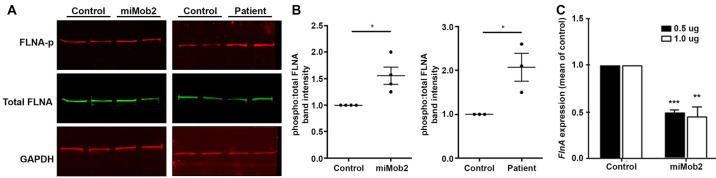
*Mob2* knockdown increases FlnA phosphorylation at Ser^2152^ *in vitro*. **(A)** C2C12 cells were transiently transfected with EGFP/empty vector control or miRNAs targeting *Mob2* (left panel). At 24 h after transfection, cell lysates were prepared and subjected to Western blot analysis using the indicated antibodies. Right panel shows results for the same experiment but performed on age-matched control or patient 1203 fibroblasts. **(B)** Quantifications representing three biological replicates of **(A)** summarizing the proportion of phosphorylated Flna at residue 2152 relative to total FLNA and normalized against the loading control Gapdh in C2C12 or age-matched fibroblast controls. Results represented as mean (± SEM). **(C)** Mean (± SEM) *FlnA* expression in C2C12 cells expressing control or *Mob2* targeting hairpins at the indicated amounts. Expression was normalized against the control using the delta-delta Ct method, as determined by real-time PCR. Expression was normalized against *Gapdh* and *Dimt1* housekeeping genes in the same sample using the relative standard curve method. Mann-Whitney U test; ^*^P < 0.05, ^**^P < 0.005, ^***^P < 0.001.

**Figure 5:**
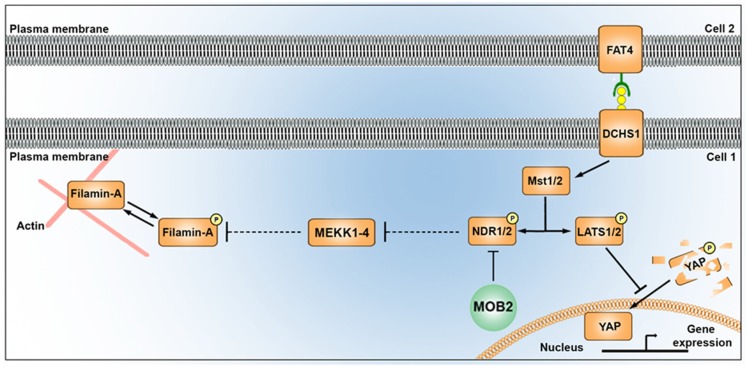
Alterations in the mammalian Hippo signaling pathway alter FLNA phosphorylation. A schematic depicting the potential link between the Hippo signaling pathway and FLNA phosphorylation as outlined in this study. MST1/2 kinases (also known as “Hippo”), activated by FAT4/DCHS1, induces phosphorylation of YAP and NDR kinases. Phosphorylation of YAP leads to its degradation in the cytoplasm, while reduced activity of MST (e.g., via loss of DCHS1 activity) attenuates YAP phosphorylation and results in its translocation in the nucleus where it promotes changes to transcription. Phosphorylation of NDR1/2 facilitates its kinase activity. NDR1/2 kinases are further modulated by MOB1 (not shown) and MOB2 either positively or negatively, respectively. NDR1/2 kinases regulate the phosphorylation of the MEKK family of proteins, known modulators of FLNA phosphorylation (at site Ser^2152^) and activity. In addition to disrupting nucleus-cilia coupling, reduced levels of MOB2 where seen in this study to induce changes in FLNA phosphorylation. A feature which could be mediated by the MEKK kinases given the associated supporting information.

### Enhanced FLNA Phosphorylation at Serine2152 After *Mob2* Knockdown *in Vitro*

In addition to regulating cilia/centrosome dynamics, Ndr1/2 kinases control actin-cytoskeletal arrangements and inhibit murine mitogen-activated protein kinases 1 and 2 (MEKK1 and MEKK2) by binding directly to residues 1169–1488 and 342–619 at their respective carboxy-termini (Enomoto et al., 2008). Interestingly, conditional depletion of *Mekk4* in the developing mouse forebrain impairs neuroependymal integrity and induces PH (Sarkisian et al., 2006). The same study also detected enhanced FlnA phosphorylation at Ser^2152^ in cells conditionally depleted of *Mekk4* (Sarkisian et al., 2006). Phosphorylation at Ser^2152^ is an important regulator of FLNA function (Loy et al., 2003). Given this insight we hypothesized a link between MOB2, NDR1/2, MEKK4 and FLNA.

Although Ndr1/2 directly binds to the carboxyl-terminus of MEKK1 and MEKK2, the study by Enomoto et al. did not investigate the binding potential of this kinase to the additional MEKK family proteins—i.e., MEKK3 and MEKK4 (Enomoto et al., 2008). Human peptide sequence alignments for this family of proteins show that the carboxyl-terminal region has the most conserved portion, with MEKK2, MEKK3 and MEKK4 having 42%, 44% and 33% sequence identity relative to MEKK1, respectively (Supplementary Figure S3). Notably, sites of highest conservation co-locate with the binding domain of NDR1/2 (Supplementary Figure S3, yellow highlight).

In light of the carboxyl-terminus conservation within the MEKK family of proteins and the reported role for Mekk4 in regulating FlnA phosphorylation at Ser^2152^ (Sarkisian et al., 2006), we next hypothesized that changes in Mob2 would alter FlnA phosphorylation at Ser^2152^. Although total FlnA protein levels were unchanged after *Mob2* knockdown in C2C12 cells *in vitro*, a significant 1.6-fold increase in FlnA phosphorylated at Ser^2152^ was observed (*P* = 0.028; Figures 4A,B). In addition, protein lysate isolated from fibroblasts of patient 1203 had a 2-fold increase in FlnA phosphorylated at Ser^2152^ relative to an age-matched control (*P* = 0.014; Figures 4A,B). *Mob2* also regulates *FlnA* mRNA expression, with a significant 2.0-fold decrease (*P* < 0.001) detected by qRT-PCR upon transfection of miRNA targeted against *Mob2* in C2C12 cells *in vitro* (Figure 4C). Together, these results suggest that altered *Mob2* expression can modulate FlnA phosphorylation and are similar to those observed upon *Mekk4* knockdown reported elsewhere (Sarkisian et al., 2006).

## Discussion

Next-generation sequencing approaches in families with two or more affected individuals have proven to be extraordinarily effective for identifying novel recessive causes of developmental conditions, including those of neurodevelopment (Yu et al., 2013; Karaca et al., 2015). In non-consanguineous families with a single affected individual (as true for the majority of patients with PH), however, this approach is difficult, and requires innovative strategies to investigate the relative contribution of candidate variants. Here, we have employed a framework that exploits reported functional interactions with loci already implicated in PH causation as a further filter through which to stratify putative biallelic candidates. Although not suggesting any given candidate satisfying this criterion will be confirmed as a contributor to PH etiology, in this study one gene—*MOB2*—was identified as having properties warranting further analysis. Functional assessment of the *MOB2* variants identified in this individual showed both to be LoF alleles. There is evidence for constraint against LoF variants in *MOB2*, as indicated by the probability of being loss-of-function intolerant (pLI) metric in the ExAC dataset being greater than 0.1 (pLI score = 0.64), as well as the very small number of LoF alleles in the gnomAD database (Lek et al., 2016). This, together with the altered cellular distribution observed after knockdown of *Mob2* within the developing mouse cortex, suggests this gene is a potential biallelic risk-conferring candidate in PH etiology with further functional and genetic analyses being warranted. However, only through the identification of more patients with *MOB2* insufficiency and PH can a direct causal association be determined.

In addition to the altered cellular distribution induced by *Mob2* knockdown, impairments in nucleus-cilia coupling were also observed. Interestingly, this phenotype was observed not only in the developing mouse cortex *in vivo*, but also in a human specific cortical development model—cerebral organoids (Lancaster et al., 2013)—suggesting that the role of Mob2 in determining cilia number and position is conserved in mammals. Given the similarities of this phenotype to that observed after *Dchs1* knockdown, a known factor of the Hippo signaling pathway and PH etiology, we hypothesize a role for cilia and MOB2 in cortical development, a prediction that is strengthened by the inability to remedy the effects of this alteration through simultaneous knockdown of Yap. While disruption of actin dynamics and cell adhesion has been a dominant theme in mechanistic studies on the pathogenesis of PH to date, the link to cilia described here could represent an unappreciated role for this organelle in the development of some forms of this particular cortical malformation.

LATS and NDR kinases are central parts of the Hippo signaling pathway in multicellular eukaryotes (Hergovich and Hemmings, 2009; Pan, 2010; Sudol and Harvey, 2010; Badouel and McNeill, 2011). Despite the importance of the NDR arm of the Hippo pathway in regulating cellular morphogenesis in eukaryotes (Kohler et al., 2010; Lin et al., 2011), the downstream targets of this kinase remain largely unknown (Ultanir et al., 2012). NDR1/2 has been experimentally identified to regulate MEKK1/2 kinase activity (Figure 5) and MEKK4 is a known regulator of FLNA phosphorylation (specifically at site Ser^2152^), a deficiency of which has been implicated in the formation of PH in the mouse cortex (Sarkisian et al., 2006; Enomoto et al., 2008). To our knowledge, a link between FLNA activity and the Hippo tumur suppressor pathway has not been described previously. Thus in this study, the association of: (1) NDR1/2 with MEKK1/2 carboxyl-terminal binding (Enomoto et al., 2008); (2) the *Mekk4*^−/−^ conditional mouse knockout inducing a PH phenotype (Sarkisian et al., 2006); and (3) the carboxyl-terminal conserved sequence between MEKK1, 2, 3 and 4 (suggesting NDR1/2 can bind and regulate all four MEKK kinases), prompted investigations into the potential link between MOB2, NDR1/2, MEKK4 and FLNA. Functional studies described here identified a contribution from MOB2 in modulating FLNA activity, suggesting a potential link between these two prominent pathways implicated in PH etiology (Figure 5). Future experiments will be required to mechanistically explore this link and indeed determine if MEKK4, or another kinase in this family, is involved. Nonetheless, FLNA phosphorylation at site Ser^2152^ has been documented to impair FLNA turnover and impairing actin cytoskeletal remodeling; features that ultimately affect neuronal migration (Sarkisian et al., 2006).

Through a framework exploiting information from known loci involved in PH, we have identified a novel candidate locus—*MOB2*—conferring potential inherited risk to PH. While functional studies and screens within larger cohorts are needed to explore this link further, we also describe a role for Mob2 in directing cortical development and present evidence that suggests that these two pathways implicated in PH etiology (i.e., FLNA and Hippo signaling) are linked.

## Author Contributions

ACO, SPR and SC conceived and designed the experiments. ACO, CK, SC and ME performed the experiments. ACO and DMM analyzed the whole-exome sequencing data. IB provided the Arl13b-tagRFP vector. MD supplied the control iPSC line HMGU1. EPK clinically evaluated the patient and provided clinical samples. MG and SPR provided funding. All authors edited and approved the manuscript.

## Conflict of Interest Statement

The authors declare that the research was conducted in the absence of any commercial or financial relationships that could be construed as a potential conflict of interest.

## Supplementary Material

### Online Resources

Allen Mouse Brain Atlas database: http://mouse.brain-map.org/

Allen Human Brain Science Atlas: http://www.brainspan.org/

Exome Aggregation Consortium (ExAC), Cambridge, MA, USA http://exac.broadinstitute.org

PubMed, https://www.ncbi.nlm.nih.gov/pubmed/

Online Mendelian Inheritance in Man (OMIM), http://www.omim.org/

1000 Genomes, http://www.1000genomes.org

Burrows-Wheeler Aligner, http://bio-bwa.sourceforge.net/

Ensembl Genome Browser, http://www.ensembl.org/index.html

GATK, http://www.broadinstitute.org/gatk/

GenBank, http://www.ncbi.nlm.nih.gov/genbank/

NHLBI Exome Sequencing Project (ESP) Exome Variant Server, ESP6500, http://evs.gs.washington.edu/EVS/

Picard, http://broadinstitute.github.io/picard/
