# Supplementary material for: Mob2 Insufficiency Disrupts Neuronal Migration in the Developing Cortex
Source: Front Cell Neurosci. 2018 Mar 12;12:57. doi: 10.3389/fncel.2018.00057 (PMC5857600; doi:10.3389/fncel.2018.00057)
Supplement: Supplementary file 1 [file Data_Sheet_1.pdf]

**Supplementary materials:**

Tables S1

Figures S1-S3

**Supplementary Table 1: List of loci with candidate loss-of-function biallelic variants identified in 65 PH trios**

| Individual | Candidate gene(s) | Chr | Position | Nucleotide | Protein | *In silico* predictions | | | |
|---|---|---|---|---|---|---|---|---|---|
| | | | | | | PolyPhen-2 | Grantham | RVIS | pLI |
| 1203 | *MOB2* | 11 | 1502019 | c.207delC | p.Phe69Phefs*127 | 1.00 (Probably damaging) | na | 24.00 | 0.64 |
| | *MOB2* | 11 | 1491530 | c.679C>T | p.Glu227Lys | 1.00 (Probably damaging) | 56 | 24.00 | |
| 2758 | *PLEKHG6* | 12 | 6422338 | c.28delG | p.Glu10Argfs*40 | 1.00 (Probably damaging) | 54 | 12.63 | 0.00 |
| 2859 | *HKDC1* | 10 | 70987024 | c.125G>A | p.Arg42Gln | 0.926 (Possibly damaging) | 43 | 70.88 | 0.00 |
| | *HKDC1* | 10 | 71000508 | IVS6-1G>A | Splice defect | 1.00 (Probably damaging) | na | 70.88 | |
| 3096 | *ARHGAP39* | 8 | 145773344 | c.24_26delTCG | p.Gln376del | 1.000 (Probably damaging) | na | 4.305 | 0.00 |
| 3495 | *CABP1* | 12 | 121093653 | c.43_44delGC | p.Ala15fs | 1.000 (Probably damaging) | na | na | 0.74 |
| | *CABP1* | 12 | 121093759 | c.146G>A | p.Arg49His | Unknown | 29 | na | |

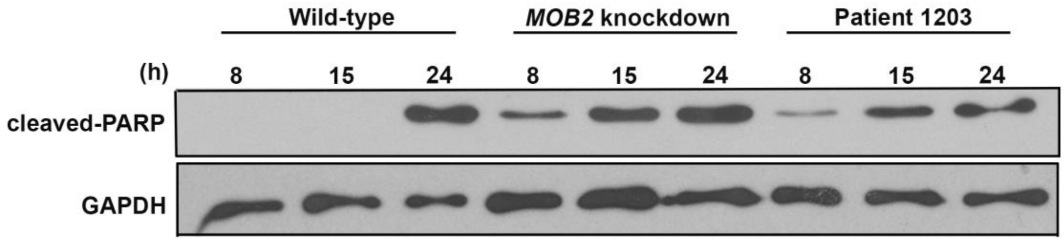


**Supplementary Figure 1: Patient 1203 fibroblasts exhibit increased susceptibility to apoptosis.** Age-matched control fibroblasts subjected to scrambled siRNA (control) or *MOB2* siRNA (*MOB2* knockdown) for 24 hours and patient 1203 fibroblasts were induced to undergo apoptosis via the addition of 200 uM camptothecin. Total cell lysates were prepared at 0, 8, 15 and 24 h post camptothecin treatment and processed for immunoblotting using the indicated antibodies.

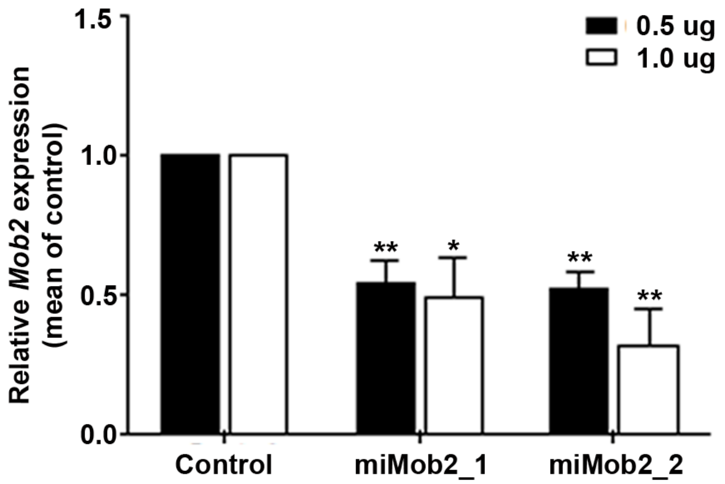


**Supplementary Figure 2: Validation of *Mob2* targeting microRNAs.** Mean (± s.e.m) *Mob2* expression in C2C12 cells 24 hours post transfection (0.5 µg or 1.0 µg) with one of two miRNA targeting *Mob2* (miMob2_1 and miMob2_2) relative to control treated sample (empty vector) expression using the delta-delta Ct method, as determined by real-time PCR. Expression was normalised against Gapdh and Dimt1 housekeeping genes in the same sample using the relative standard curve method.Mann Whitney U test; * $P < 0.05$, ** $P < 0.005$.

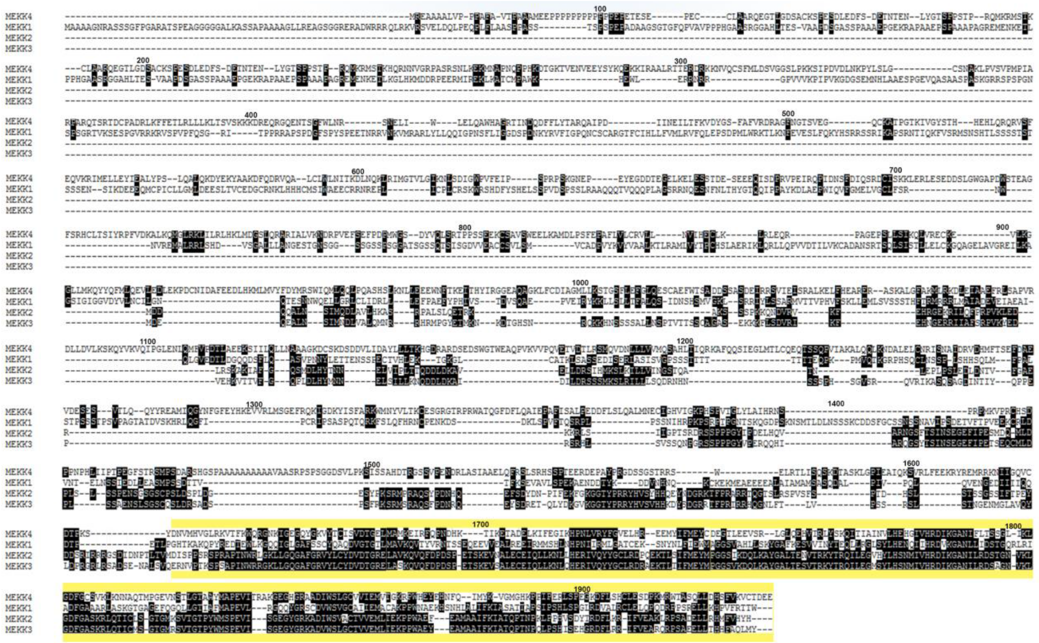

**Supplementary Figure 3: Carboxy-termini conservation among MEKK family proteins.** Peptide sequence conservation among MEKK family proteins, MEKK1-4. Detailed residue alignments with sites of at least 50% conservation among the four proteins highlighted in black. The C-terminal peptide sequence containing NDR1/2 binding domain identified in common between MEKK1 and MEKK2, as defined by Enomoto *et al*., (*1*) is flanked by yellow.

## Reference:


1. A. Enomoto *et al.*, Negative regulation of MEKK1/2 signaling by serine-threonine kinase 38 (STK38). *Oncogene* **27**, 1930-1938 (2008).
